# Sural Nerve Size in Fibromyalgia Syndrome: Study on Variables Associated With Cross-Sectional Area

**DOI:** 10.3389/fmed.2020.00360

**Published:** 2020-07-24

**Authors:** Marco Di Carlo, Claudio Ventura, Pietro Cesaroni, Marina Carotti, Andrea Giovagnoni, Fausto Salaffi

**Affiliations:** ^1^Rheumatology Clinic, Ospedale “Carlo Urbani”, Università Politecnica delle Marche, Ancona, Italy; ^2^Radiology Department, Ospedali Riuniti, Università Politecnica delle Marche, Ancona, Italy

**Keywords:** sural nerve, fibromyalgia, ultrasound, body mass index, neuropathic pain features

## Abstract

Increased cross-sectional area (CSA) of sural nerve, documented by ultrasound (US), has been revealed in small fibers neuropathy, condition present in about half of patients with fibromyalgia (FM). The aims of this study were to evaluate sural nerve CSA and to establish the variables associated with increased CSA in FM patients. A cross-sectional assessment was conducted in consecutive FM patients. Demographic data, clinimetric parameters [Fibromyalgia Impact Questionnaire (FIQR)], the neuropathic pain features [PainDetect Questionnaire (PDQ)], and the sural nerve CSA were recorded. CSA was determined by US, examining the sural nerve at the lateral region of the calf. CSA was compared with demographic and clinical variables. A multiple regression analysis was conducted applying CSA as dependent variable. One hundred and ten FM patients were enrolled. Sural nerve CSA showed a significant association with body mass index (BMI) (*r* = 0.422; *p* < 0.0001) and with PDQ (*r* = 0.361; *p* = 0.0001). The multiple regression analysis confirmed that BMI (*p* = 0.0001) and PDQ (*p* = 0.0028) were the two independent variables associated with CSA. The severity of the disease, measured with FIQR, showed no association. An increase in sural nerve CSA is closely related to BMI and to distinctive neuropathic symptoms. Overweight and obesity appear to be associated with a FM phenotype with documented peripheral nervous system involvement. Ultrasound examination of the sural nerve at calf level may reveal useful information in patients with FM, identifying a cluster of patients with peripheral nervous system alterations. This cluster of patients is generally overweight or obese, and complains of painful symptoms with neuropathic features.

## Introduction

Fibromyalgia syndrome (FM) is a condition characterized by the presence of chronic widespread pain (CWP) associated with somatic symptoms, the latter mainly characterized by the presence of fatigue, memory impairment, and non-restorative sleep. These symptoms are those that define FM according to the latest set of criteria proposed by the American College of Rheumatology (ACR) for diagnostic/classification purposes (1). The clinical burden is such that the quality of life of patients with FM is worse than that of other rheumatic diseases (2). Trying to define the pathogenesis of the complex symptomatology has been an object of intense research in recent years. However, it is far from being possible to ascertain a unique pathophysiological mechanism that can explain the symptoms in an unequivocal way, and there are still no biomarkers useful for diagnostic purposes (3). Pathophysiological mechanisms involving the central nervous system (CNS) have been documented. Magnetic resonance imaging studies have revealed the presence of volume reduction in gray matter regions of pain processing, as well as alterations in descending pathways of pain modulation and functional connectivity (4). Alongside the presence of changes at the CNS level, pathological modifications have also been documented at the level of the peripheral nervous system (PNS). In particular, several studies have documented the presence of small fibers neuropathy (SFN) in a significant percentage of patients with FM (5). The prevalence of SFN appears to be around 40–49% in FM patients (6, 7). The presence of SFN can be demonstrated by histological examination performed on skin biopsy aimed at determining the intra-epidermal nerve fibers density (IENFD) or by examination of corneal innervation by confocal microscopy. Through the latter technique it has been demonstrated that alterations of the small fibers are present in about half of all FM patients (8).

Overall this finding, namely that a SFN is present in about half of all FM patients, has been confirmed by a recent meta-analysis that grouped all the studies that used either skin biopsy or cornea confocal microscopy (6). However, to date, it is difficult to refer all FM patients to perform a skin biopsy or a complex ophthalmological evaluation.

An interesting work, performed on patients with SFN but outside of FM, has documented how SFN is associated with an increase in the cross-sectional area (CSA) of the sural nerve compared to healthy volunteers (9). The sural nerve is a small nerve with sensory function and, from the ultrasound (US) point of view, is an easily accessible structure. The current last-generation ultrasound machines also allow an excellent definition of nerve structures, even if they are millimetric in size (10). In the field of peripheral nerve diseases, ultrasound (US) is increasingly accepted diagnostic technique and innovative fields of research in this area are opening up (11).

Starting from these considerations, the aims of this were to study sural nerve CSA with US in FM patients, to identify the variables associated with and to identify the predictive variables of increased sural nerve CSA.

## Materials and Methods

### Setting and Patients

In the present study, from June 2019 to December 2019, patients affected by FM were consecutively enrolled in a third-level rheumatology center that represents the regional referral for the diagnosis and treatment of FM. The diagnosis of FM, formulated according to the 2016 ACR criteria (1), was defined in all patients by FS, a rheumatologist with over 30 years experience in the management of FM. Demographic data were collected for each patient, in particular weight and height, in order to calculate the body mass index (BMI), comorbidities, and current drug therapy.

Only adult patients were considered for the purposes of this study, regardless of the severity state of the disease. Patients with known neurological diseases affecting the PNS or CNS (i.e., patients suffering from polyneuropathies, Parkinson's disease, Alzheimer's disease, or other dementias), patients with internistic of rheumatological diseases that may lead to the involvement of the small nerve fibers (patients suffering from type II diabetes mellitus, chronic renal failure, uncontrolled endocrinopathies, ongoing neoplasms, HIV-HBV-HCV infections, vasculitides, or connective tissue diseases, patients with current or previous use of drugs inducing neuropathies), and patients with conditions that may interfere with clinical evaluation (e.g., concomitant inflammatory arthropathies in a phase of high disease activity or severe symptomatic osteoarthritis) were excluded. Patients who have previously undergone small saphenous vein stripping for the treatment of varicose veins have also been excluded, given the possibility, albeit remote, that this procedure may have induced damage to the sural nerve (12).

All patients agreed to participate in the study by signing informed consent, and the procedures conducted in the study were approved by the local Ethics Committee (Comitato Unico Regionale—ASUR Marche, *n*. 1970/AV2).

### Clinimetric Assessment

Patients underwent a clinical evaluation to identify the severity of the disease, measured through the revised Fibromyalgia Impact Questionnaire (FIQR), and to identify neuropathic pain features through the use of the PainDetect Questionnaire (PDQ). The clinimetric evaluation was conducted at the rheumatology visit, and was carried out by a clinical fellow in rheumatology (PC) with experience in the administration of questionnaires.

The main features of the two questionnaires are as follows. The FIQR is realized by 21 numerical rating scales with 11 points, where the score 10 indicates for the worst symptomatology, is a completely patient-reported tool, and explores three health domains (symptoms−10 items, function−9 items, overall impact−2 items) through questions related to the last 7 days. The final score is given by the algebraic sum of the three domains, where the score of the 10 symptom items is divided by two, the score of the 9 function items is divided by three, and the two overall impact items are considered as they are. The final score ranges from 0 to 100, where higher scores identify a higher severity of disease (13).

PDQ has been validated in several contexts, including FM, as a fully patient-reported instrument for measuring the neuropathic features of painful symptoms (14). It does not require an objective examination and is based solely on the symptomatology reported by the patient. The questions (seven items with 5-point scales, with the anchors “never,” score 0, and “very strongly” score 5) are oriented on neuropathic symptoms such as sudden pain, allodynia, dysesthesia, and hyperalgesia. There is also a manikin where the patient has to indicate the irradiated pain (irradiated pain is scored 2 points), and four items (of which the patient has to choose only one) that describe the temporal pattern (score from −1 to +1 depending on the indicated temporal pattern). The overall score ranges from −1 to 38, to be interpreted as a low probability of the presence of neuropathic pain (<15%) for scores <12, and a high probability (>90%) of the presence of neuropathic pain for scores >19. The range between 13 and 18 defines an ambiguous result (15).

### Ultrasound Assessment of the Sural Nerve

US examination of the sural nerve was conducted by two experts sonographers, respectively, MD (a rheumatologist with 10 years of experience in musculoskeletal US) and CV (a radiologist with 5 years of experience in musculoskeletal US). Both operators were blinded to the clinical and clinimetric evaluation carried out in each patient.

The agreement on how to conduct the US examination was obtained through a 4-h preliminary training session where the two physicians assessed together 10 healthy subjects and 10 FM patients (not considered in the final analysis) under the supervision of MC, a musculoskeletal radiologist with over 20 years of experience in US. At the end of the preliminary training both operators were able to recognize the sural nerve independently, and to measure its circumference and CSA.

**Figure 1:**
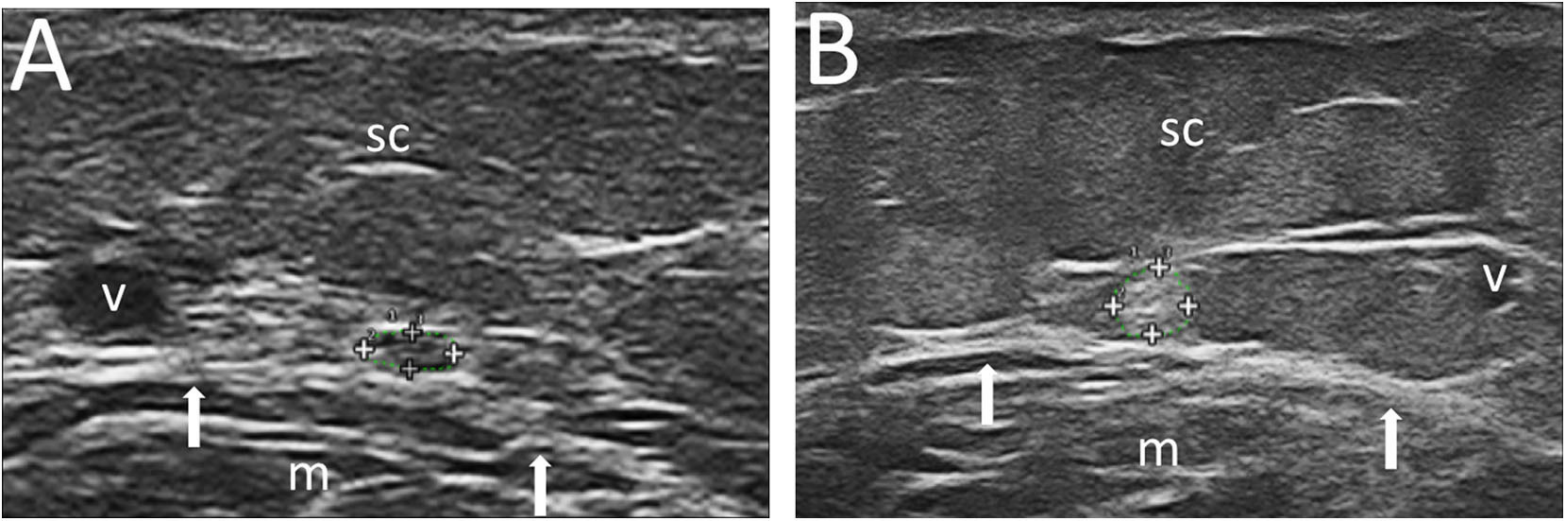
Ultrasound images of the sural nerve at the lateral and distal portion of the calf. In **(A)**, sural nerve CSA 1 mm^2^ in a 54-year-old woman, with BMI = 20.0 Kg/m^2^ and PDQ = 9. In **(B)**, sural nerve CSA = 8 mm^2^ in a 32-year-old woman, with BMI = 35.9 Kg/m^2^, and PDQ = 25. Also note in **(B)** the greater representation of subcutaneous tissue. Legend: sural nerve contoured within the + signs; v, small saphenous vein; m, gastrocnemius muscle; arrows, gastrocnemius muscle margin; sc, subcutaneous tissue.

With reference to the execution technique, the US examination was carried out with the patient in prone decubitus, with his feet out of the bed. Before starting the US examination, 14 cm in the posterior region of the calf from the apex of the malleolus were recorded as the point of reference for the ultrasonographic measurements. At this level the sural nerve is a small structure posterior to the flat tendon of the gastrocnemius muscle and is detectable in the immediate proximity of the small saphenous vein (Figure 1). This latter structure is easily identified by US (10).

The decision to measure at this level was taken from what has already been described in a work on peripheral nervous involvement in systemic vasculitides (16). Measurements were taken freehand on the freeze images, and the circumference of the sural nerve (expressed in mm) and its CSA (expressed in mm^2^) were measured bilaterally, three times on each side (considering the mean value of the three measurements for each parameter). The US examination was completed by the power Doppler study of the sural nerve and perineural regions to identify possible inflammatory signs.

MD performed the US using a MyLab Class C (Esaote S.p.A, Genoa, Italy) equipped with a 6–18 MHz multifrequency broad band probe, while CV used an Epiq 5 (Philips) equipped with a 4–18 MHz multifrequency broad band probe.

### Statistical Analysis

The data of this study are presented with descriptive statistics as mean and standard deviation (SD).

The correlations between the variables were studied through Pearson's rank correlation test. This analysis was conducted primarily to verify the correlation between circumference and CSA.

To verify the objective of the study, in particular to identify the association of the studied variables with sural nerve CSA, a one-way analysis of variance (ANOVA) with *post-hoc* test for pairwise comparisons (Scheffe's test), was firstly conducted. Then, to investigate the variables (independent variables) associated with increased sural nerve CSA (dependent variable), a multiple regression analysis was conducted. Among the independent variables were considered demographic variables such as age and BMI, and clinical variables, including disease duration, disease severity (measured as FIQR score), neuropathic pain features (assessed through PDQ), and treatment (including in this variable patients taking pregabalin, duloxetine, or a combination of these two drugs). The collinearity between independent variables was considered for *r* > 0.9 at correlation analysis. As dependent variable, the mean value of CSA between the right and left side of the body was considered.

Since the US measurements were carried out by two separate operators, the inter-reader reliability was calculated through the intraclass correlation coefficient (ICC) on a sample of patients evaluated by both sonographers.

Significant values of *p* were considered <0.05. Statistical analyses were performed using MedCalc 18.0.0.

## Results

The study was conducted on 110 patients, respectively, 105 women (94.5%) and 5 men, with a mean age (±SD) of 51.02 (±11.20) years. The mean duration of FM (±SD) was 5.76 (±5.23) years. With regard to pharmacological treatment, 64 patients (58.2%) were treated with pregabalin (at a dosage of at least 150 mg/day), 54 patients (49.1%) were treated with duloxetine (60 mg/day), of these 28 patients (25.5%) were on pregabalin + duloxetine combination therapy.

The mean value of BMI was 28.07 (± 6.08) Kg/m^2^. Respectively, 43 patients (39.09%) had a normal BMI (between 18.5 and 24.9 kg/m^2^), 26 patients (23.63%) were overweight (BMI between 25.0 and 30 kg/m^2^), while 41 patients (37.27%) were obese (BMI above 30.1 kg/m^2^).

Regarding the severity of the disease, the mean score (±SD) for FIQR was 64.17 (±19.01). The presence of neuropathic pain features was also found to be high, with a mean PDQ score (±SD) of 20.48 (±6.62). Respectively, in 66 patients (60.0%) a high probability of neuropathic pain features was documented, in 30 patients (27.3%) an ambiguous result was found, while in 14 patients (12.7%) a low probability of neuropathic pain features was revealed.

US examination of the sural nerve documented a mean circumference (±SD) of 6.92 (±6.70) mm and a mean CSA of 2.94 (±2.52) mm^2^. Using the power Doppler technique, the presence of a pathological hyperemia has not emerged in any studied nerve.

Table 1 summarizes the main clinical and demographic characteristics of the population studied.

**Table 1:** Demographic, clinimetric, and sural nerve features of the population sample (110 patients).

	**Mean**	**Standard deviation**
Age (years)	51.02	11.20
Disease duration (years)	5.76	5.23
BMI (Kg/m^2^)	28.07	6.08
FIQR	64.17	19.01
PDQ	20.48	6.62
Circumference (mm)	6.92	1.56
CSA (mm^2^)	2.94	1.27

*BMI, Body Mass Index; FIQR, revised Fibromyalgia Impact Questionnaire; PDQ, PainDetect Questionnaire; CSA, cross-sectional area*.

The correlations results (Table 2) showed that a high correlation between the mean circumference and mean CSA measurements (*r* = 0.945, *p* < 0.0001) was detectable in the evaluation of sural nerve dimensions. Both measurements correlated significantly with BMI (*r* = 0.422; *p* < 0.0001) and PDQ (*r* = 0.361; *p* = 0.0001). A slight correlation, at the limits of significance was found between CSA and FIQR (*r* = 0.208; *p* = 0.02).

**Table 2:** Correlation table (Pearson's *r*) among the variables studied.

		**Circumference**	**CSA**	**Disease duration**	**Age**	**FIQR**	**PDQ**
BMI	Correlation coefficient *r*	0.450	0.422	0.054	0.161	0.166	0.227
	Significance level *p*	< 0.0001	< 0.0001	0.57	0.09	0.08	0.01
Circumference	Correlation coefficient *r*		0.945	−0.120	0.102	0.180	0.362
	Significance level *p*		< 0.0001	0.21	0.29	0.06	0.0001
CSA	Correlation coefficient *r*			−0.093	0.06	0.208	0.361
	Significance level *p*			0.33	0.53	0.02	0.0001
Disease duration	Correlation coefficient *r*				0.136	0.065	−0.049
	Significance level *p*				0.15	0.49	0.61
Age	Correlation coefficient *r*					0.023	−0.020
	Significance level *p*					0.81	0.83
FIQR	Correlation coefficient *r*						0.556
	Significance level *p*						< 0.0001

*BMI, Body Mass Index; CSA, cross-sectional area; FIQR, revised Fibromyalgia Impact Questionnaire; PDQ, PainDetect Questionnaire*.

**Figure 2:**
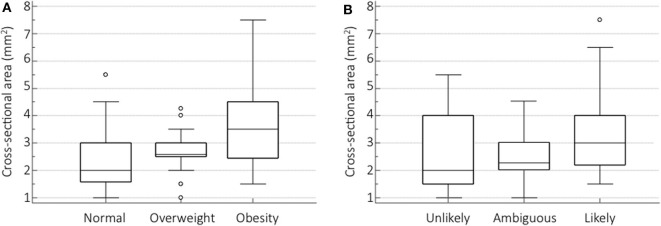
Sural nerve CSA differences for BMI and PDQ categories. Box-and-whisker plots showing the sural nerve CSA differences among BMI categories **(A)** and PDQ **(B)** categories measured by one-way analysis of variance (ANOVA) with *post-hoc* test for pairwise comparisons (Scheffe's test).

Among the variables studied through the ANOVA, the only two variables associated with sural nerve CSA were BMI and PDQ. Both BMI categories (*p* < 0.001) (Figure 2A) and PDQ categories (*p* = 0.003) (Figure 2B) showed statistically significant differences in relation to sural nerve CSA.

Multiple regression analysis confirmed that the only two independent variables significantly associated with sural nerve CSA were BMI (*p* = 0.0001) and PDQ (*p* = 0.0028), while patient age, disease duration, treatment and even FIQR score did not showed any association (Table 3). Removing the large proportion of obese patients from the analysis, in the 69 normal weight or overweight subjects PDQ became the independent variable most associated with CSA (*p* = 0.0038), followed by FIQR (*p* = 0.0263) and BMI (*p* = 0.0432) (Table 4).

**Table 3:** Multiple regression analysis of the independent variables related to the cross-sectional area (dependent variable) of the sural nerve.

**Independent variables**	**Coefficient**	**Standard error**	***t***	***p***	***r_**partial**_***	***r_**semipartial**_***
Constant	−0.5261					
BMI	0.08358	0.01987	4.207	0.0001	0.3862	0.3573
Disease duration	−0.01769	0.02436	−0.726	0.4695	−0.07206	0.06167
Treatment	0.007743	0.021321	0.675	0.8087	0.009872	0.001231
Age	0.0006697	0.01070	0.0626	0.9502	0.006227	0.005316
FIQR	−0.002547	0.007306	−0.349	0.7280	−0.03467	0.02962
PDQ	0.06569	0.02140	3.069	0.0028	0.2921	0.2607

*BMI, Body Mass Index; FIQR, revised Fibromyalgia Impact Questionnaire; PDQ, PainDetect Questionnaire*.

**Table 4:** Multiple regression analysis of the independent variables (same variables as Table 3) related to the cross-sectional area (dependent variable) of the sural nerve, excluding obese patients.

**Independent variables**	**Coefficient**	**Standard error**	***t***	***p***	***r_**partial**_***	***r_**semipartial**_***
Constant	0.7051					
BMI	0.07385	0.03577	2.064	0.0432	0.2536	0.2326
Disease duration	0.001606	0.02159	0.0744	0.9409	0.009448	0.008380
Treatment	0.007896	0.02332	0.680	0.8189	0.009992	0.001452
Age	−0.004673	0.01028	−0.455	0.6510	−0.05763	0.05120
FIQR	−0.01614	0.007088	−2.277	0.0263	−0.2778	0.2565
PDQ	0.06209	0.02065	3.006	0.0038	0.3567	0.3386

*BMI, Body Mass Index; FIQR, revised Fibromyalgia Impact Questionnaire; PDQ, PainDetect Questionnaire*.

The ICC was calculated on 26 patients evaluated by both sonographers. The inter-reader agreement was excellent for both the circumference (ICC = 0.97, 95% confidence interval 0.94–0.98) and the sural nerve CSA measurement (ICC = 0.96, 95% confidence interval 0.92–0.98).

## Discussion

To the best of our knowledge, this is the first study that evaluated sural nerve size by US in patients with FM. BMI and neuropathic pain features, estimated as PDQ score, are the two clinical variables associated with and predictive of sural nerve CSA. Our results could have implications in the identification, through an easily repeatable and non-invasive technique, of a cluster of FM patients with PNS involvement. In turn, the identification of this cluster of patients could lead to an improvement in the diagnostic/classification aspects. Currently the diagnosis of FM, with various differences between the various sets of criteria, remains however, an umbrella term that mainly encompasses the concept of CWP and therapeutic aspects.

It is well-known that peripheral nerves may reveal an increased CSA at the site of compressive neuropathies or in case of polyneuropathies with different etiologies (10). However, some work suggests that the size of some nerves may be an expression of what happens to small nerve fibers. In their interesting paper of 2015, Ebadi et al. demonstrated that sural nerve CSA was increased also during SFN. The pathophysiology of how a SFN induces morphological changes in the sural nerve must be clarified, but it is assumed that an augmented CSA is an expression of alterations in axoplasmic flow involving the small diameter A delta and C fibers (9). In FM patients, it can be assumed that increased sural nerve CSA may be an expression of SFN.

The pathogenesis of FM is still a long-standing research topic, and the study of small nerve fibers is a recent area of interest. Giannoccaro et al. for the first time in 2014 introduced skin biopsy in FM patients, revealing the presence of SFN in six of the 20 patients studied (5). Since then, several studies have followed until a recent meta-analysis that documented the presence of a SFN in about half of the patients with FM (6).

On the other hand, the diagnosis of SFN is a constantly evolving field. To date, the evaluation of IENFD by skin biopsy is the commonly accepted practice in the international literature. However, the significance of identifying SFN in FM patients is much debated, and some authors question the value of skin biopsy since the variability of IENFD is wide, and histological signs of SFN are present even in a significant proportion of healthy subjects (17).

In the present study we considered sural nerve CSA as a surrogate for SFN, revealing its close association with BMI. The association between SFN and obesity was recognized over 10 years ago. The mechanisms involved in the damage to small nerve fibers in obese subjects would seem to be attributable to poorly regulated glucose control mechanisms and excessive production of oxidants (18).

Other studies have previously revealed that peripheral nerves can be increased in the obese population. In these subjects, a study conducted at the median nerve level, revealed that CSA of the nerve itself is increased in asymptomatic obese subjects with normal carpal pressure. The authors concluded that this finding could be related to metabolic causes (19).

The size of peripheral nerves can also be influenced by other variables, one of which is age. A previous work showed a correlation between peripheral nerve CSA and age (20). In our study this correlation did not emerge, and probably due to the fact that the size of the sural nerve is influenced less than other nerves by age (21). Body weight itself is also another factor that can affect peripheral nerve CSA. However, compared to other nerves, sural nerve CSA is the one that seems to be less affected by this variable (22).

Also the link between obesity and FM has been known for years, although the cause-effect relationship (i.e., whether obesity comes first or whether obesity may be a consequence of physical inactivity) has not yet been clarified (23). About 75% of FM patients have a change in body composition that leads them to have more fat mass, resulting in an increased perception of painful symptoms, fatigue, stiffness, and reduced quality of life (24, 25). Obesity *per se* represents a condition for which more inflammatory mediators are produced, which can lead to a sensitization of painful symptoms (26), and probably a significant proportion of this sensitization depends on damage to small nerve fibers.

The correlation between sural nerve CSA and PDQ can be intuitive and expected. The fact that an US expression of PNS damage correlates with the PDQ score, enhances the role of this questionnaire in the assessment of FM patients. The use of questionnaires aimed at investigating the presence of neuropathic pain features has been criticized in FM patients because it is believed that high scores depend on impaired central pain control (27). In our study we demonstrated how the data from a patient-reported questionnaire, investigating the presence of neuropathic components of pain, are effectively correlated with US measurements. This fact can corroborate that the use of PDQ remains valid, especially in a category of patients suffering from FM and overweight or obesity.

The lack of association with parameters that identify the impact of disease (i.e., FIQR) leads us to affirm that sural nerve CSA is not an expression of the severity of the disease but rather, it can identify a category of patients with major PNS involvement.

Finally, we have shown that physicians with experience in musculoskeletal US can, with a short training, also learn to perform US of the sural nerve in an extremely reliable way.

The main limitation of the study is represented by the fact that in this cohort of patients was not performed the histological examination on skin biopsy, the diagnostic test considered the gold standard to document the presence of a SFN. In a future research it will certainly be necessary to explore the correlation between histological and US diagnosis also in FM patients. Another potential limitation is recruitment to a single center. However, the US examination of the sural nerve was carried out by two independent operators, demonstrating that, after a short training period, an excellent agreement can be reached using different machines. Another limitation of the study was not including a healthy control group. In that case, however, it would have been difficult to apply the definition of healthy subject in obese individuals and thus compare the role of BMI in patients and controls.

Sural nerve CSA measured with a simple US examination could be a very useful parameter for the identification of a subpopulation of FM patients with NPS involvement. It is believed that this type of involvement is more prevalent in overweight/obese subjects and in those who have painful symptoms with neuropathic features. Confirmation of the results of this study should be obtained in future studies where the data from the US examination can be integrated with the histological findings.

## Data Availability Statement

The raw data supporting the conclusions of this article will be made available by the authors, upon reasonable request to the corresponding author.

## Ethics Statement

The studies involving human participants were reviewed and approved by Comitato Etico Unico Regionale. The patients/participants provided their written informed consent to participate in this study.

## Author Contributions

MD, FS, MC, and AG conceived the study. MD, CV, and PC were involved in data collection. MD and FS analyzed and interpreted data. MD was the major contributor in writing the manuscript. All authors read and approved the final manuscript.

## Conflict of Interest

The authors declare that the research was conducted in the absence of any commercial or financial relationships that could be construed as a potential conflict of interest.
